# Whole-Genome Analysis, Biosynthetic Gene Cluster Mining of *Amycolatopsis* sp. nov. TRM77291 and Antibacterial Activity of Siderochelin A

**DOI:** 10.3390/microorganisms14092096

**Published:** 2026-09-19

**Authors:** Yi-Huang Chen, Lu-Yao Wang, Tao Luo, Mei-Ting Wang, Yu-Xiang Yao, Jian-Ming Wang, Zhu-Feng Zhang, Xiao-Xia Luo

**Affiliations:** Key Laboratory of Protection and Utilization of Biological Resources in Tarim Basin of Xinjiang Production & Construction Corps, College of Life Science, Tarim University, Alar 843300, China

**Keywords:** *actinobacteria*, taxonomic identification, secondary metabolites

## Abstract

The genus *Amycolatopsis*, belonging to the phylum Actinobacteria, is known for producing diverse bioactive secondary metabolites and plays a significant role in microbial drug discovery. This study centers on the strain TRM77291, sourced from a unique habitat, and is postulated to represent a potential new species. This hypothesis is based on 16*S rRNA* gene phylogenetic analysis combined with physiological, biochemical, and chemotaxonomic characterization. The strain’s genome, consisting of 8.76 Mb with a G + C content of 68.89% and encoding 8572 genes, was analyzed through whole-genome sequencing. AntiSMASH analysis identified 38 biosynthetic gene clusters related to PKS, NRPS, and RiPPs, while BIG-SCAPE analysis indicated the potential for unique compound synthesis, including Cluster 7 and Cluster 35. Screening ten fermentation media determined millet medium as the most optimal choice. Siderochelin A, a monomeric compound isolated from a fermentation product, demonstrated antibacterial efficacy against various pathogenic bacteria. This study revealed a notable activity of Siderochelin A against *Escherichia coli*, providing a theoretical basis for discovering novel species, exploring secondary metabolites, and developing innovative antibiotics.

## 1. Introduction

*Amycolatopsis* is a Gram-positive aerobic bacterium known for its abundant mycelium production [1,2]. It belongs to the order *Actinomycetales*, family *Pseudonocardiaceae*. Initially classified within the *Streptomyces* genus, it was later reassigned to *Nocardia* before being designated as a genus of anamorphic mycobacteria in 1986 due to its distinct cell-wall composition and resistance to Nocardia-specific phage. Currently, the genus comprises 99 species and five subspecies as of 2023 [3].

Many *Amycolatopsis* strains exhibit a robust capacity for secondary metabolism, producing a diverse array of secondary metabolites with notable bioactive properties [4]. The secondary metabolites fall into various categories, including polyphenols, linear polyketides, macrolides, cyclic peptides, glycopeptides, amides, amino derivatives, and glycoside derivatives, showcasing their antimicrobial, cytotoxic, and antitumor activities [5]. *Amycolatopsis*, an important antibiotic-producing genus, synthesizes significant metabolites such as (1) glycopeptide antibiotics like vancomycin, which itself has served as a key clinical anti-Gram-positive agent since the 1950s [5]; (2) polyketide antibiotics like rifamycin, chelerythrine, tobramycin, and conomycin A; and (3) the polyene antibiotic chelocardin initially isolated from *A. sulphurea* in the 1970s now garnering attention for its efficacy against drug-resistant bacteria [6]. *Amycolatopsis* also produces eremomycin (from *A. orientalis* INA 238) and other bioactive compounds. The structural diversity and potent antimicrobial activity of these metabolites position them as a valuable resource for clinical therapeutics and the development of novel antibiotics [7].

The genus *Amycolatopsis* has been extensively investigated, yet strains inhabiting specialized environments may harbor untapped metabolic capabilities. Here, the strain TRM77291 was isolated from river-bottom sludge of the Tarim River Basin and identified as a novel species. Analysis using antiSMASH and BIG-SCAPE revealed 38 secondary metabolic gene clusters, including Cluster 7 and Cluster 35, with the potential for synthesizing novel active compounds. Fermentation optimization led to the isolation and identification of Siderochelin A, demonstrating antimicrobial activity against *E. coli* for the first time. This study aims to elucidate the taxonomic classification and metabolic profile of acidophilic bacteria in this distinct habitat, offering insights for the development of novel antibiotics.

## 2. Methods

### 2.1. Strain Preservation

The experimental strain TRM77291 was isolated from Tarim River silt and conserved by the microbiology group of Tarim University and the Conservation Center for Typical Cultures of China under CCTCC M 2024327. *Amycolatopsis speibonae* (TRM70016), *Amycolatopsis lurida* (TRM64344), and *Amycolatopsis thailandensis* (TRM70032), as well as pathogenic bacteria *Acinetobacter baumannii*, *Candida albicans*, *Staphyloccocus aureus*, *Pseudomonas aeruginosa*, and *Escherichia coli*, were preserved by the microbiology group of Tarim University.

### 2.2. Identification of Strains

The genomic DNA of the strain was extracted using the CTAB method [8]. The 16*S rRNA* gene was amplified with universal primers 27F (5′-AGAGTTTGATCCTGGCTC-3′ and 1492R (5′-CGGCTACCTTGTTACGACTT-3′). The obtained sequences were assembled using SeqMan softwareversion 7.1.0.44 and deposited in GenBank to acquire their accession numbers. Multiple-sequence alignment was performed using the EzBioCloud database (https://www.ezbiocloud.net/, accessed on 21 April 2025), and the phylogenetic tree was constructed with MEGA X [9]. The test strain was compared with phylogenetically related strains from the same niche. Whole-genome sequences of the test strain and related reference strains were submitted to the ANI web server (https://www.ezbiocloud.net/tools/ani, accessed on 21 April 2025) and the GGDC 3.0 web server (http://ggdc.dsmz.de/ggdc.php, accessed on 21 April 2025) for genomic distance calculation.

### 2.3. Morphological Characteristics, Physiological and Biochemical Characteristics, and Analysis of Cellular Chemical Components

Strain TRM77291 was inoculated onto ISP series media, Gao’s No. 1 medium, and Czapek-Dox medium. The cultures were incubated at 30 °C for 7 days. Morphological characteristics, including substrate mycelium, aerial mycelium, and pigment production, were observed. For scanning electron microscopy (SEM) analysis, TRM 77291 was cultured in ISP4 liquid medium at 28 °C with shaking at 150 rpm for 2–3 days. Cells were harvested by centrifugation at 6000 rpm for 5 min under 4 °C. The cell pellets were fixed using 2.5% glutaraldehyde, followed by gradient ethanol dehydration and critical-point drying. Micrographs were acquired with a JSM-6360 scanning electron microscope JEOL Ltd. [10]. Physiological characteristics of the strain were determined by examining its growth at temperatures ranging from 4 to 50 °C, pH values of 4–11, and NaCl concentrations of 0–25% (*w*/*v*). Enzyme activities (including peroxidase and urease) and other physiological properties, such as carbon source utilization and gelatin liquefaction, were assayed following standard protocols [11,12]. For chemotaxonomic analysis, whole-cell sugars in cell-wall hydrolysates were detected, and polar lipids were separated and identified using two-dimensional thin-layer chromatography (2D-TLC) [13,14].

### 2.4. Analysis of Metabolic Potential of the Genome of Strain TRM77291

The complete genome sequences of strain TRM77291 and similar strains were uploaded to antiSMASH (https://antismash.secondarymetabolites.org, accessed on 7 September 2026) for secondary metabolite biosynthesis gene cluster prediction [15].In order to clarify the specificity of secondary metabolite capacities for TRM77291 and similar strains, the biosynthetic gene clusters of TRM77291 and similar strains were summarized with the help of antiSMASH and were analyzed in comparison with each other, whereas the metabolic potentials of the studied strain and these similar strains were analyzed by BIG-SCAPE [16].

### 2.5. Fermentation Optimization and Activity Screening of Strain TRM77291

Strain TRM77291 was inoculated at an inoculum ratio of 1% (*v*/*v*) onto ten different fermentation media, with the corresponding blank media used as controls. Detailed information on the ten media is provided in Supplementary Table S1. The biomass was freeze-dried, extracted with an equal volume of methanol, and then sonicated in an ultrasonic crusher for 2 h. The extract was concentrated by rotary evaporation, and then subjected to HPLC detection (HPLC conditions: column—GIST C18 (4.6 mm × 250 mm, 5 μm); mobile phase—methanol–water from 10 to 100% methanol, 40 min; 100% methanol, 10 min; detection wavelength—190–800 nm; column temperature—40 °C; flow rate—1 mL/min; injection volume—10 µL). The pathogenic bacteria were incubated on MHA medium for 12 h. The plate activity assay was performed, and the activity of the strains was judged according to the inhibition diameter [17].

### 2.6. Isolation and Structural Analysis of the Fermentation Products of Strain TRM77291

Strain TRM77291 was cultivated in 70 L millet medium (15.0 g boiled millet, 5.0 g glucose, 4.0 g peptone, 3.0 g NaCl, and 16.0 g agar per 1000 mL distilled water at pH 7.0) in a 100 L fermenter and incubated at 30 °C for 10 days. The fermentation broth was spray-dried to yield 504.8 g of powder. The obtained methanol extracts were combined and concentrated under reduced pressure at 60 °C. The resulting crude extract was dissolved in 0.25 L of ultrapure water and loaded onto an MCI-gel CHP20P reversed-phase resin column. Step gradient elution was performed using 0%, 30%, 50%, 70%, and 100% MeOH-H_2_O solutions, with each gradient applied for three column volumes. Fractions were collected at 200 mL per fraction, yielding five fractions designated A, B, C, D, and E. Purification of sub-fractions C2–C6 was performed under bioactivity-guided fractionation. The compounds were dissolved in deuterated methanol, and the isolated products were analyzed using nuclear magnetic resonance spectroscopy, such as ^1^H-NMR and ^13^C-NMR, and mass spectrometry.

### 2.7. Validation of Monomer Compound Activity of Strain TRM77291

In order to detect the biological activity of the single compound, the antagonistic activity of the compound against *Escherichia coli* ATCC 25922 was tested in this experiment using the filter paper sheet diffusion method at concentrations of 512, 256, 128, 64, 32, 16, 8, 4, 2, and 1 μg/mL, and the MIC assay was carried out using a 96-well plate [18].

## 3. Results

### 3.1. Determination of the Genetic Classification of the Strain

#### Molecular Characteristics

**Figure 1:**
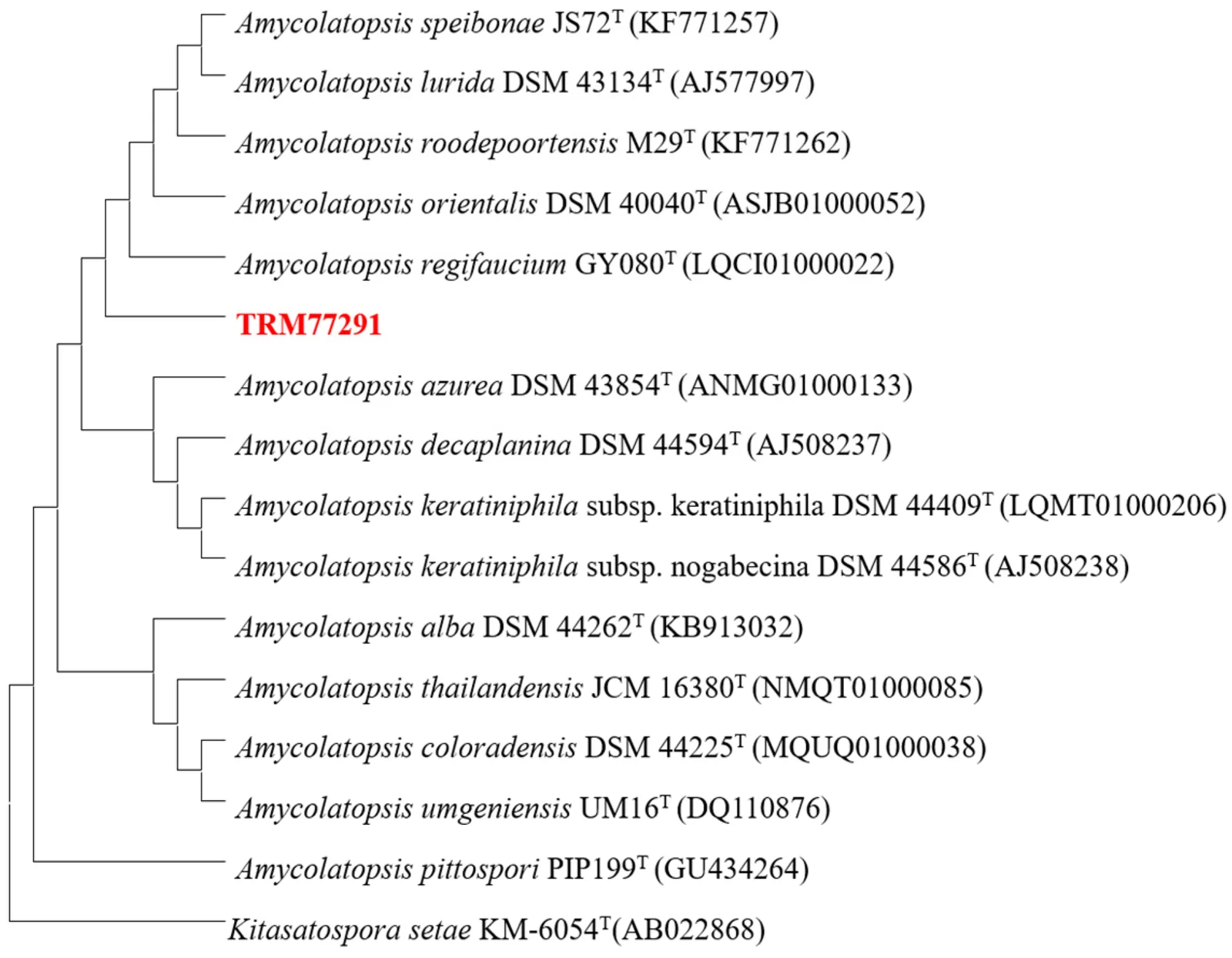
Phylogenetic tree of *16S rRNA* gene of strain TRM77291 and similar strains.

The 16*S rRNA* gene of strain TRM77291 exhibited a 99.49% similarity to the most closely related strain, *Amycolatopsis speibonae*, followed by *Amycolatopsis lurida*, with 99.35% similarity. A phylogenetic tree was constructed for strain TRM77291, showing it as a distinct branch (Figure 1). Subsequent second-generation sequencing analysis revealed an Average Nucleotide Identity (ANI) value of 93.93% and a DNA–DNA Hybridization (DDH) value of 66.50% between TRM 77291 and *A. speibonae*, as well as an ANI value of 93.93% and a DDH value of 58.50% between TRM77291 and *A. lurida*. These values fall within the thresholds for interspecies differentiation (new species if ANI is <95%, and different species of the same genus if DDH is 60–70%). Therefore, strain TRM 77291 is proposed as a potential new species of *Amycolatopsis speibonae*.

### 3.2. The Morphological, Physiological, and Biochemical Characterization of Strain TRM77291

Strain TRM77291 was cultured in various media for 7 days under identical conditions to assess its growth and morphological characteristics, as detailed in Table 1. The strain exhibited robust growth on Gao’s No. 1 and Czapek-Dox medium media; good growth on ISP1, ISP2, ISP4, and ISP7 media; and weak growth on ISP3 and ISP5 media. Aerial mycelia were absent on the ISP1 and ISP6 media, whereas purple pigmentation was observed exclusively on Gao’s No. 1 medium.

**Figure 2:**
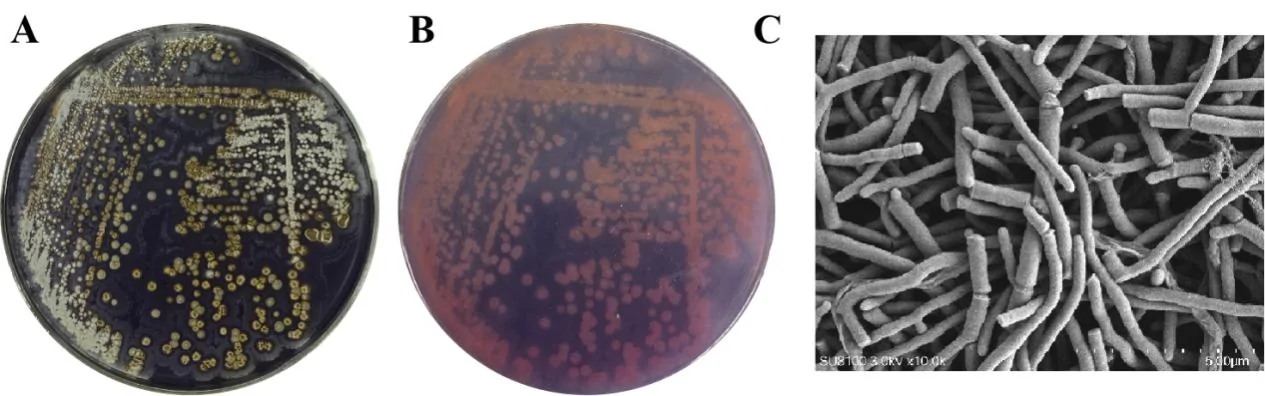
Morphological characterization of strain TRM77291. Note: (**A**) presents the plate growth morphology of strain TRM77291 (front); (**B**) presents the plate growth morphology of strain TRM77291 (back); and (**C**) shows an electron micrograph of strain TRM77291.

Strain TRM77291 was cultured on Gao’s No. 1 medium at 30 °C for 7 days. Electron microscopy revealed well-developed yellow aerial mycelium and abundant production by the strain (Figure 2A,B). The mycelium exhibited a compact distribution, and the spore chain morphology resembled bamboo rods with a smooth surface, as depicted in Figure 2C.

Strain TRM77291 exhibited a growth range of 4–40 °C, with optimal growth at 37 °C; it tolerated sodium chloride concentrations of 0–5% and pH levels of 7–8, with its peak growth at pH 7.0. It utilized various carbon sources, including galactose, lactose, glucose, xylose, inositol, D-cellulose, sorbitol, L-arabinose, D-mannitol, maltose, and mannan oligosaccharide, for growth. The strain demonstrated milk peptonize; gelatin liquefaction; nitrate reduction; the decomposition of Tween 20, Tween 40, Tween 60, and Tween 80; and lipase production. However, it lacked the ability to hydrolyze starch, decompose cellulose, or produce hydrogen sulfide or melanin. Detailed physiological and biochemical characteristics of strain TRM77291 are presented in Table 2 alongside similar genera for comparison.

### 3.3. The Chemical Characterization of Strain TRM77291

**Figure 3:**
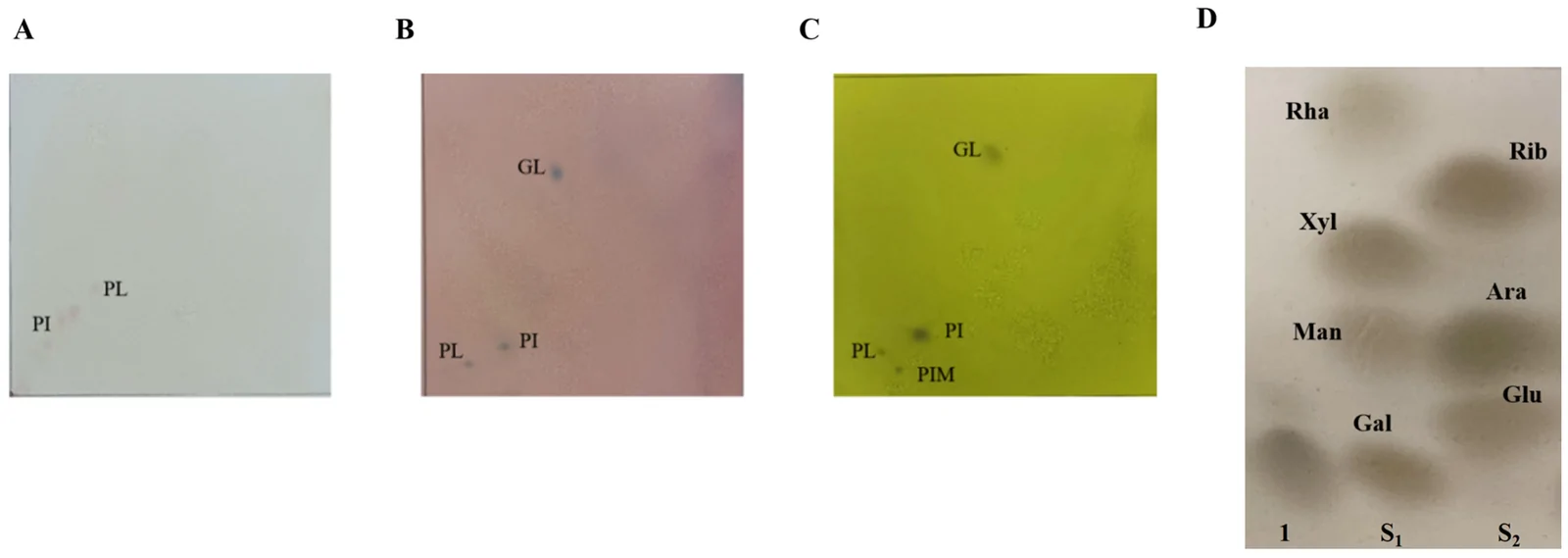
Chemical indicators of strain TRM77291. Note: Biphasic TLC color development of polar lipids from strain TRM77291—(**A**) anisaldehyde color development, (**B**) ninhydrin color development, and (**C**) molybdenum phosphate color development; phosphatidylinositol (PI), phosphatidyl-myo-inositol marmoside (PIM), phospholipid (PL), glycolipid (GL). (**D**) is the hydrolyzed whole-cell sugar profile of strain TRM77291—Rha is rhamnose, Rib is ribose, Xyl is xylose, Ara is arabinose, Man is mannose, Glu is glucose, and Gal is galactose; 1 is TRM77291 hydrolyzed whole-cell sugar, and S1 and S2 are sugar markers.

The primary constituent of TRM77291’s hydrolyzed whole-cell sugar was galactose, as shown in Figure 3. The predominant cellular fatty acids, each comprising over 5% of the total, were iso-C15:0 (22.23%), iso-C16:0 (14.19%), C17:9 (12.92%), anteiso-C17:0 (5.29%), and C18:0ω9c (8.14%). Analysis using three chromogenic agents indicated that the cell membrane of strain TRM77291 consisted of Phosphatidylinositols (PIs), phosphatidyl-myo-inositol marmosides (PIMs), Phospholipids (PLs), and glycolipids (GLs), as depicted in Figure 3.

Strain TRM77291 exhibits distinct molecular, physiological, biochemical, and chemical traits compared to its counterparts, suggesting its classification as a novel species within the genus *Amycolatopsis*.

### 3.4. Genomic Analysis and Metabolic Potential Mining of Strain TRM77291

Whole-genome sequencing of strain TRM77291 revealed a genome size of 8.76 Mb with a G + C content of 68.89%. The strain harbors a total of 8572 genes, comprising 73 tRNAs, five rRNAs, and one tmRNA.

#### 3.4.1. Predicted Biosynthetic Gene Clusters of Strain TRM77291

The whole-genome sequence of strain TRM77291 was analyzed using antiSMASH, revealing the presence of 38 biosynthetic gene clusters, including PKS, NRPS, Terpene, RiPP, hydrogen cyanide, Arylpolyene, and ectoine classes. Further details can be found in Table 3.

Notably, Cluster 19 is responsible for encoding aminopolycarboxylic acid metal carriers; Cluster 33 resembles the NAPAA gene cluster, with 19% similarity to pyridomycin biosynthesis; and Cluster 34 is an NRP-metallophore gene cluster sharing 78% similarity with the mirubactin biosynthetic gene cluster. These clusters are associated with various iron carrier compounds, suggesting the potential of strain TRM77291 to synthesize such compounds.

The strain’s secondary metabolic capacity was assessed using antiSMASH, revealing a concentration in the PKS, NRPS, and RiPP classes. Furthermore, by comparing gene cluster similarity and completeness levels with known clusters, several clusters with potential were identified. For example, Cluster 7 showed potential to synthesize a structure similar to MDP, while Cluster 35 showed promise in producing the lasso peptide Albusnodin.

#### 3.4.2. Comparative Genomic Analysis of Strain TRM77291 in Comparison with Genetically Similar Strains

**Figure 4:**
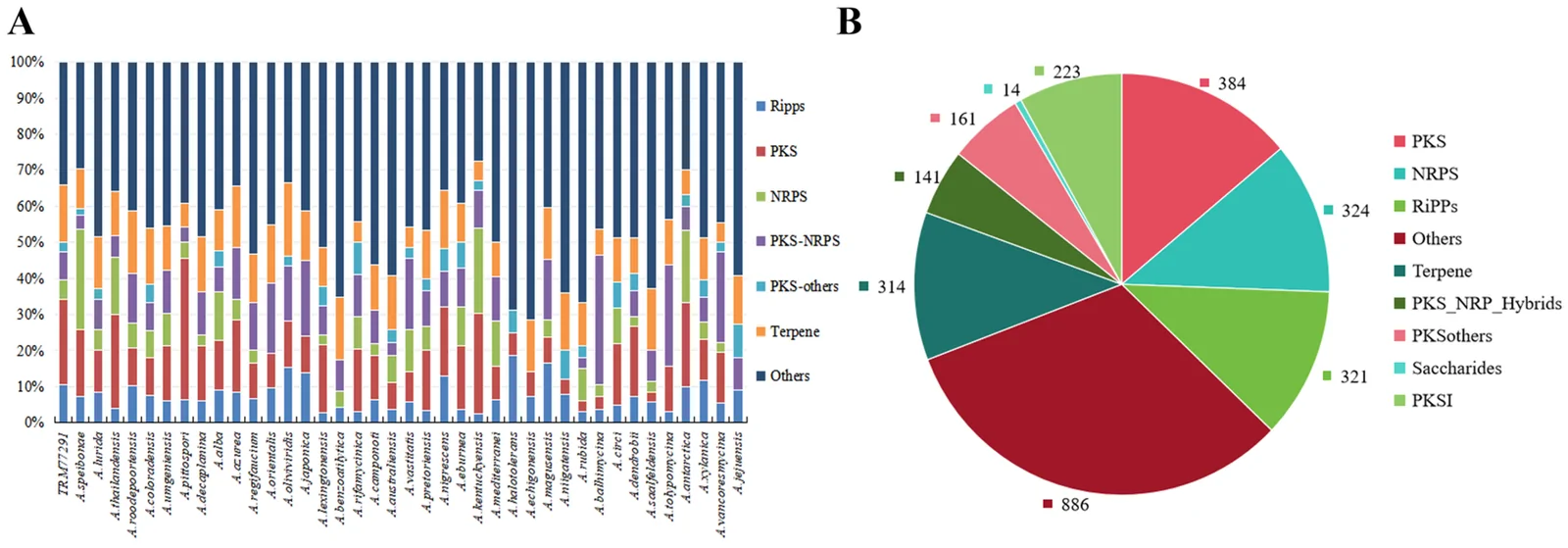
(**A**) Distribution of gene clusters in the genomes of TRM77291 and its similar strains; (**B**) distribution of biosynthetic gene clusters in *Amycolatopsis*.

The biosynthetic gene clusters of strain TRM77291 were analyzed and compared with those of 39 similar strains (refer to Table 1). The analysis revealed that *A. anthracis* exhibited a relatively abundant secondary metabolism capacity. The gene cluster responsible for synthesizing PKS-like secondary metabolites in strain TRM77291 was notably predominant within its overall metabolic capacity, ranking second only to the strains of *A. thailandensis*, *A. pittospori*, and *A. kentuckyensis*. Notably, Cluster 35 was identified as a unique gene cluster in strain TRM77291, distinguishing it from other mycobacteria and indicating its specific secondary metabolism capabilities (Figure 4).

#### 3.4.3. In-Depth Analysis of the TRM77291 Biosynthetic Gene Cluster Using Bigscape

The Bigscape analysis of *Amycolatopsis anisopliae* strains revealed a diversified secondary metabolic potential within this genus. Specifically, a total of 314 families were found to encode terpenoids, while 84 families encoded PKSs, 324 families encoded NRPSs, and 321 families encoded RiPPs. Additionally, 886 unclassified biosynthetic gene cluster family types were identified, as depicted in Figure 4. These findings suggest that the genus harbors significant potential for bioprospecting.

**Figure 5:**
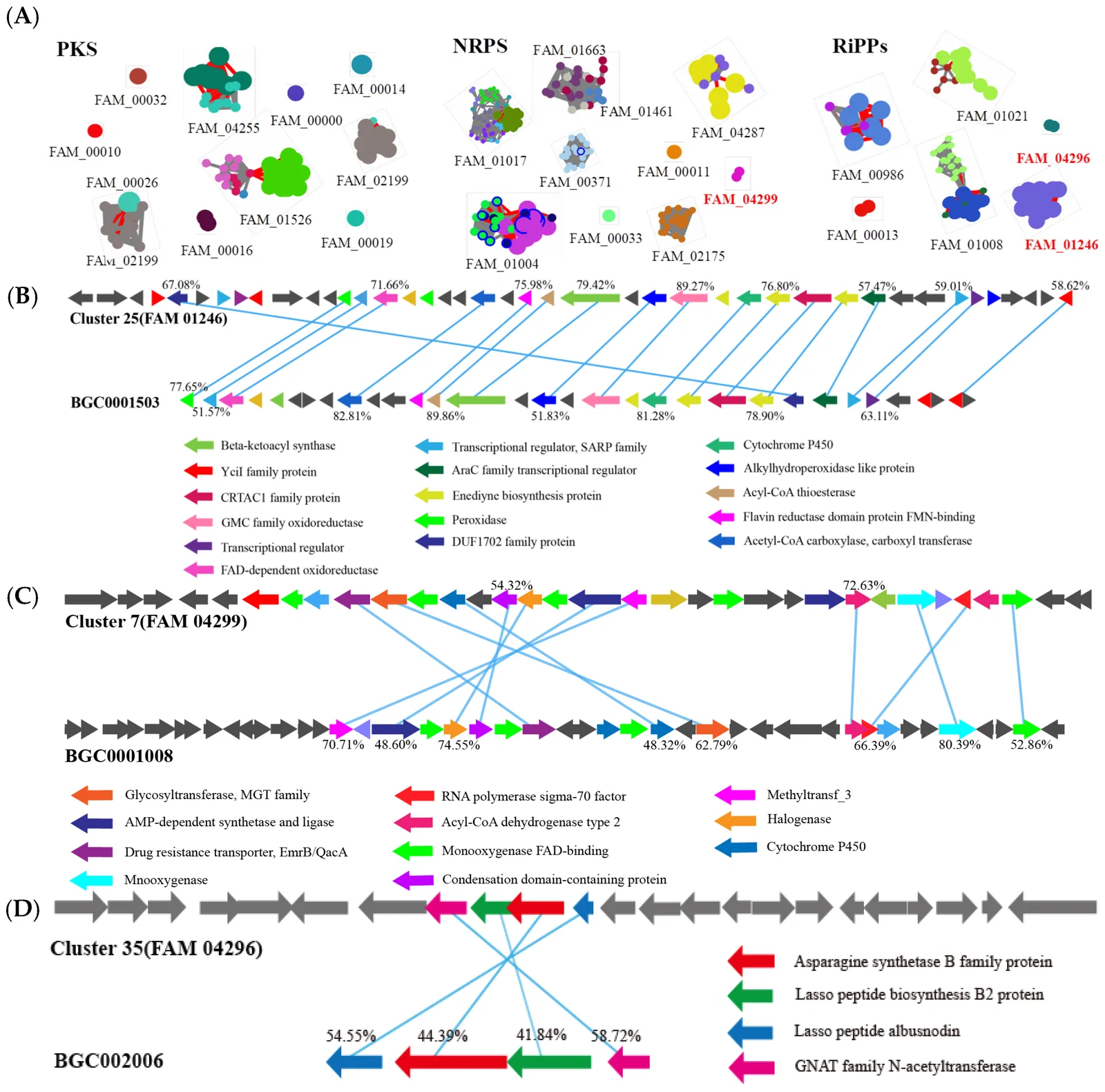
The results of BIG-SCAPE analysis of the gene clusters of *Amycolatopsis*. Note: (**A**) shows the results of BIG-SCAPE analysis (molecular network diagram); (**B**) shows the comparative analysis of Cluster 25 with similar gene clusters; (**C**) shows the comparative analysis of Cluster 7 with similar gene clusters; and (**D**) shows the comparative analysis of Cluster 35 with similar gene clusters.

The BIG-SCAPE analysis of strain TRM77291 identified a biosynthetic gene cluster responsible for PKS-like natural product synthesis, as shown in Figure 5A. No distinct biosynthetic gene cluster of significant research value was individually identified. Cluster 25 of this strain, belonging to the RiPP class, showed 31% similarity to a known gene cluster for Amycolamycin A/B synthesis, as seen in Figure 5B. Comparative analysis indicated the potential for other related compound synthesis. Through these clusters, an NRPS-like natural product synthesis in strain TRM77291 showed 39% similarity to the Maduropeptin (MDP) biosynthesis gene cluster. Core genes related to MDP synthesis were present in Cluster 7 (Figure 5C), suggesting the potential for anti-tumor natural product synthesis similar to MDP.

FAM 04296 represents a distinct node in the molecular network diagram The family corresponds to Cluster 35 of strain TRM77291, spanning from 438,023 to 460,583 bp in the genome sequence, with a total length of 22,561 bp. Cluster 35 exhibits 50% similarity to the gene cluster associated with Albusnodin synthesis. A comparison between Cluster 35 and its closest gene cluster, BGC 002006, reveals that the genes within Cluster 35 share core gene similarities with BGC002006, along with additional functional gene fragments, including ctg80_444 and ctg80_455, which are involved in transcriptional regulation. Figure 5D and Table 4 demonstrate the potential of this gene cluster to produce novel natural products.

Through individual analyses of strain gene clusters following the BIG-SCAPE global analysis, two potential gene clusters, namely Cluster 7 and Cluster 35, were ultimately pinpointed. These clusters exhibit a comparable structure in the biosynthesis of MDP and the lasso-peptide-type compound Albusnodin.

### 3.5. OSMAC Screening Based on Activity and Metabolites

**Figure 6:**
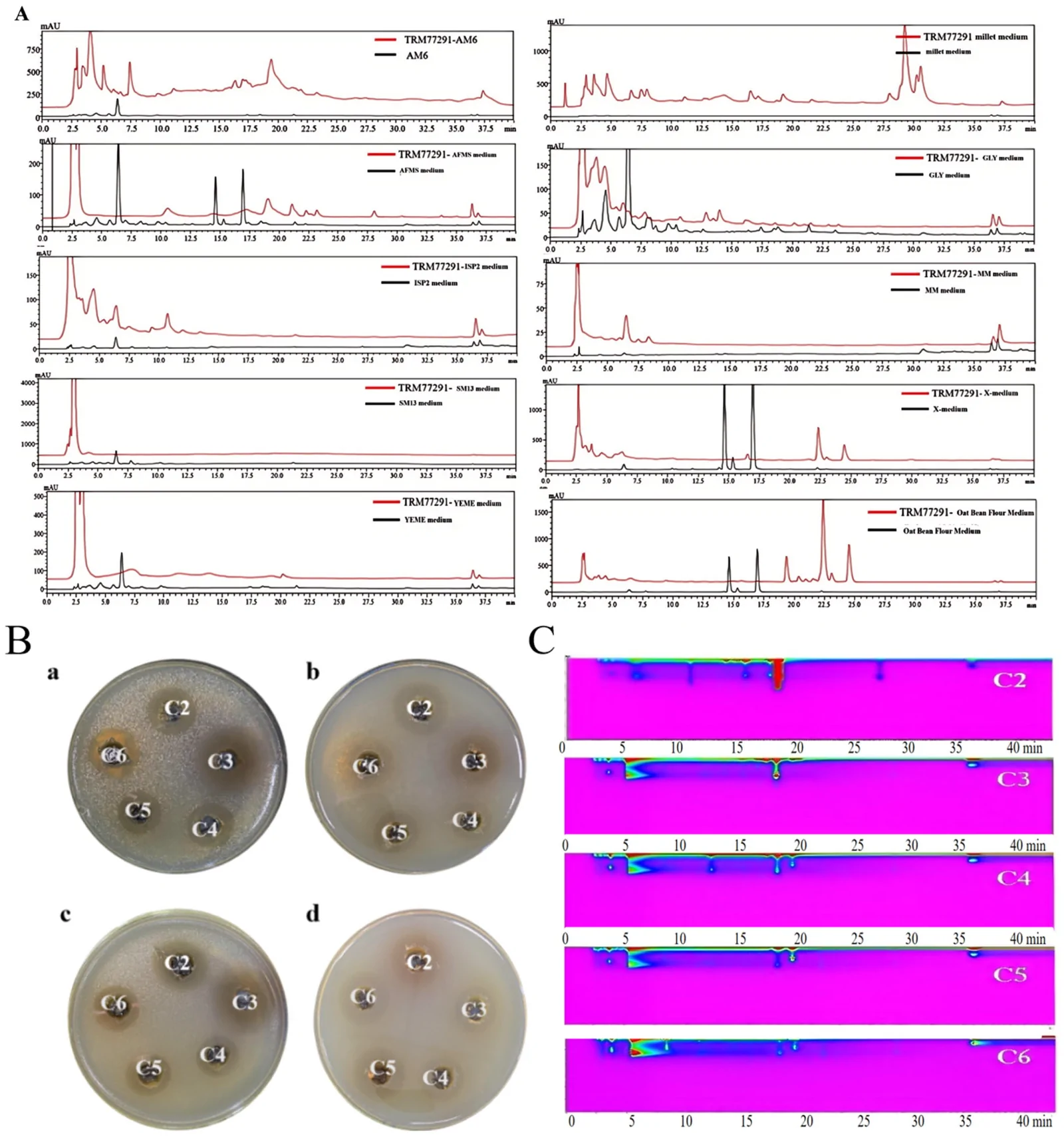
(**A**) HPLC profiles of TRM77291 fermentation crude material from different fermentation media; (**B**) bacteriostatic activities after column chromatography; (**C**) HPLC profiles of the active fractions. Note: (**B**) presents the image of the antagonistic activities of five components C2–C6 against different pathogens ((**a**) is the graph of inhibitory activity of the five components against *Escherichia coli*; (**b**) is the graph of inhibitory activity of the five components against *Pseudomonas aeruginosa*; (**c**) is the graph of inhibitory activity of the five components against *Eruinia amylovora*; (**d**) is the graph of inhibitory activity of the five components against *Salmonella*); (**C**) shows the graphs of liquid-phase assay of the five components C2–C6.

The studied strain exhibited a high production of secondary metabolites within enriched media, such as millet medium, AM6 medium, X fermentation medium, AFMS medium, modified GLY medium, and oat–soy flour medium. However, in the oligonutrient MM medium, strain TRM77291 did not demonstrate any significant production of secondary metabolites, as illustrated in Figure 6A.

The activities of the fermentation product extracts from ten different fermentation media were assessed. The results revealed that only the crude extracts of millet fermentation media exhibited antagonistic activities against *Acinetobacter baumannii*, *Candida albicans*, *Staphylococcus aureus*, *Pseudomonas aeruginosa*, and *Escherichia coli*. The specific antagonistic performances are detailed in Table 5.

The use of millet medium enhanced the production of secondary metabolites with increased activity for strain TRM77291. Following activity testing experiments, the strain TRM77291 was chosen for fermentation in millet medium. The active fractions demonstrated inhibitory activities against *L. amyloliquefaciens*, *Salmonella*, *E. coli*, and *Pseudomonas aeruginosa* within the C2–C6 fractions. The HPLC profiles and specific activities are illustrated in Figure 6B,C.

### 3.6. Identification and Activity Verification of Compound Activity

**Figure 7:**
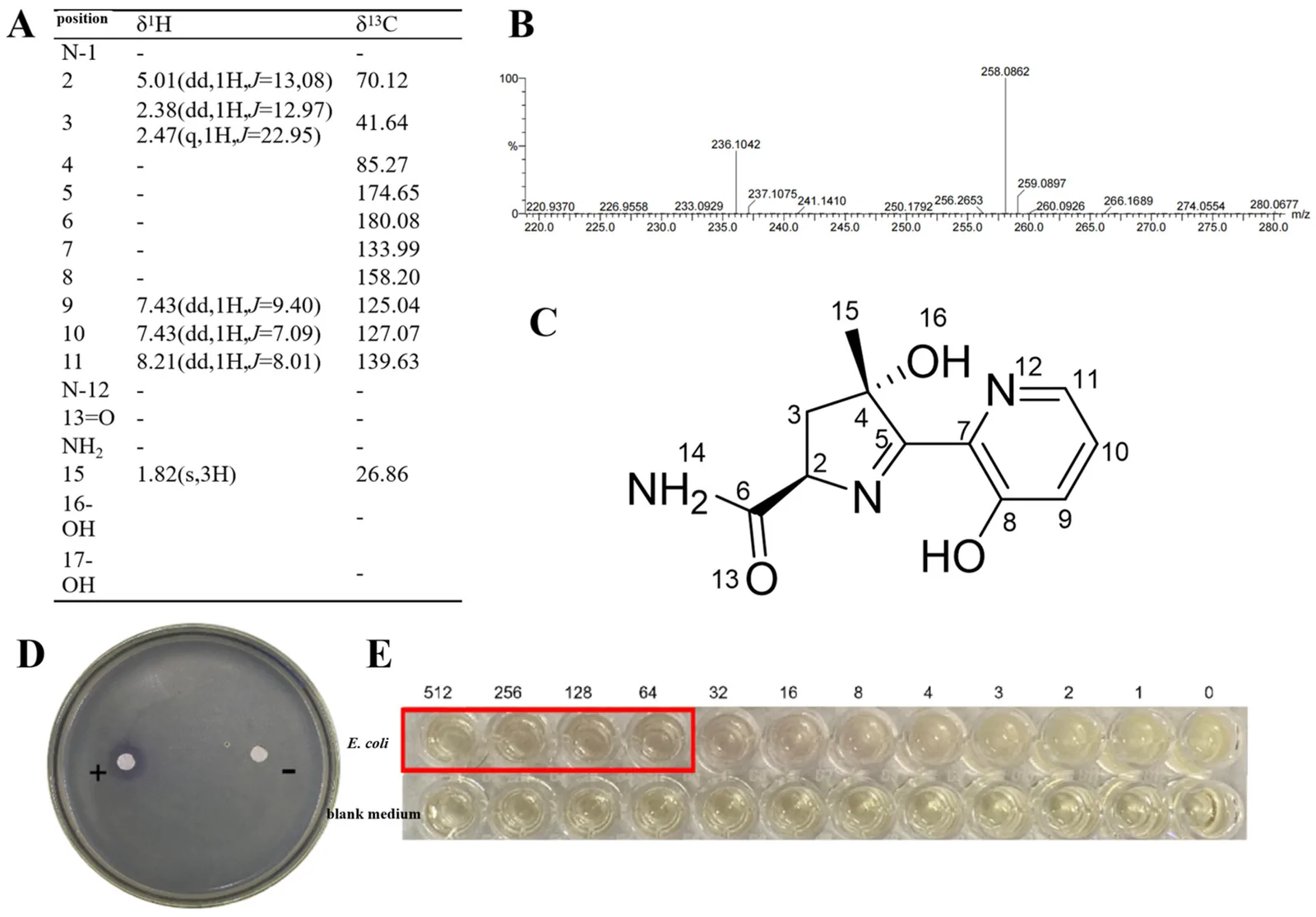
Compound 77291-C1 structural identification information and 77291-C1 activity plot against *E. coli* CCTCC 25922. Note: (**A**) shows the hydrocarbon attribution information of compound 77291-C1, (**B**) shows the mass spectra of compound 77291-C1, and (**C**) is the structure of compound 77291-C1. (**D**) shows the image of antagonistic activities of compounds against the target bacteria using the filter paper sheet diffusion method; + (1 mg/mL Siderochelin A, with methanol as the solvent); - (methanol); the target bacteria is *Escherichia coli* CCTCC 25922. (**E**) The minimum inhibitory concentrations (MICs) of the compound against the target bacteria, as determined by the micro two-fold dilution method, with concentrations in μg/mL.

During the concentration of a single fraction sample under reduced pressure, crystals were observed in the C2 fraction. Upon drying and subsequent addition of an appropriate quantity of dichloromethane, yellowish crystals precipitated at the bottom of the vial, from which compound monomer 77291-C1 was isolated as a crude mixture yielding 1040 mg. Comparison of the ^13^C NMR data with published microspectroscopic data indicated a close match with the carbon and hydrogen spectral data of Siderochelin A. (Figure 7A–C). The compound’s molecular mass was confirmed through mass spectrometry analysis, with HR Q-TOF MS data of the compound (m/z 236.1042 [M + H]+, 258.0862 [M + Na]+) aligning with these values (m/z 236.1313 [M + H]+, 258.1138 [M + Na]+) (Figure 7B). The molecular formula was determined to be C_11_H_13_N_3_O_3_ [19], and the compound structure is depicted in Figure 7, with the supporting spectra provided in Figure 1 and Figure 2.

Siderochelin A, as reported in the literature, exhibits antagonistic activity [20]. In our study, these compounds were found to possess bacteriostatic effects against *E. coli*, with a zone of inhibition of 11 ± 3 mm (Figure 7D). Additionally, concentrations ranging from 64 to 256 μg/mL exhibited bacteriostatic activity against *E. coli* CCTCC 25922, with a minimum inhibitory concentration (MIC) of 64 μg/mL, indicating moderate antibacterial activity (Figure 7E).

## 4. Discussion

The taxonomic and functional analysis of *Amycolatopsis* sp. nov. TRM77291 enhances understanding of the genus’ phylogenetic and metabolic diversity. TRM77291 shows a 16*S rRNA* gene similarity of 99.49% to *A. speibonae* and ANI/DDH values below the species thresholds (93.93% and 66.50%, respectively), indicating its classification as a novel species according to current genomic criteria for prokaryotic classification [21]. Its genome, comprising 8.76 Mb with a G + C content of 68.89%, contains 38 biosynthetic gene clusters (BGCs), including the distinction of Cluster 7 (39% similarity to Maduropeptin) and Cluster 35 (50% similarity to Albusnodin), setting it apart from other known species [22]. This genomic flexibility reflects patterns in actinobacterial evolution, where BGC diversity is influenced by specific habitats [23]. Functionally, TRM77291 produces Siderochelin A [20], exhibits antibacterial activity against *Escherichia coli* (MIC = 64 μg/mL), and expands the compound’s efficacy beyond mycobacterial inhibition, whose original structural elucidation was reported in 1981 [20]. This discovery highlights the genus’ potential for novel antibiotic development, especially considering the strain’s unique BGCs and growth preferences (optimal at 37 °C, pH 7.0). These findings are in line with current drug-discovery strategies, emphasizing the importance of actinobacteria from specialized environments in combating antimicrobial resistance [24].

## 5. Conclusions

The strain TRM77291 was identified as a potential new species of the genus *Amycolatopsis* through polyphasic taxonomic analyses. Its *16S rRNA* gene exhibited 99.49% similarity with *A. speibonae*, although its ANI (93.93%) and DDH (66.50%) values fell below species-defining thresholds. With a genome size of 8.76 Mb, it harbored 38 clusters of secondary metabolic genes, including endemic Cluster 7 (39% similarity to Maduropeptin) and Cluster 35 (50% similarity to Albusnodin). Fermentation optimization revealed that crude extracts from millet media displayed broad-spectrum activity against five pathogenic bacteria. This is the first report of Siderochelin A production by an *Amycolatopsis* species isolated from the Tarim Basin and its antibacterial activity against *E. coli*.

## Figures and Tables

**Table 1:** Summary of the morphological characteristics of strain TRM77291.

Medium	Growth	Intracellular Mycelium	Aerial Mycelium	Pigmentation
ISP1	++	-	White	-
ISP2	++	White	Light yellow	-
ISP3	+	White	White	-
ISP4	++	White	Light yellow	-
ISP5	+	Light yellow	White	-
ISP6	+	-	Translucent yellow	-
ISP7	++	White	Light yellow	-
Gao’s No. 1	+++	White	Yellow	Purple
Czapek-Dox medium	+++	White	White	-

Note: +++ means that the strain grew vigorously; ++ means that the strain grew well; + means that the strain grew poorly; - means that the strain did not grow or was not produced.

**Table 2:** Physiological and biochemical profiles of TRM77291 and its closely related strains.

	TRM77291	1	2	3
Galactose	++	++	++	++
Lactose	+++	+++	+++	+++
Glucose	+++	+++	+++	++
Xylose	+++	+++	+++	+++
Inositol	+++	+++	+++	+++
D-Cellobiose	+++	+++	+++	+++
L-Arabinose	+++	+++	+++	+++
Maltose	+++	+++	+++	+++
Mannan Oligosaccharide	+++	+++	+++	++
Sorbitol	++	++	++	++
Temperature Tolerance °C	4–40	4–40	4–40	4–40
Optimum Temperature °C	37	30	30	30
Salt Concentration Range %	0–5	0–5	0–5	0–5
pH Range	7–8	7–11	6–8	7–8
Tween 20	+	+	+	+
Tween 40	++	++	++	++
Tween 60	+++	+++	+++	+++
Tween 80	+	+	+	+
Starch Hydrolase	−	−	−	−
Cellulase	−	−	−	−
Melanin	−	−	−	−
Hydrogen Sulfide	−	−	−	−
Milk Coagulation and Peptonization	Coagulation	Coagulation	Coagulation	Coagulation
Nitrate Reduction	+	+	+	+
Gelatin Liquefaction	+	−	+	−

Note: 1—*Amycolatopsis speibonae*, 2—*Amycolatopsis lurida*, 3—*Amycolatopsis thailandensis*. +, weak positive reaction; ++, moderate positive reaction; +++, strong positive reaction; −, negative reaction.

**Table 3:** Summary of gene cluster prediction results for the strain TRM77291.

Cluster	Type	Location	Most Similar Known Cluster	Similarity
1	T1PKS	1-4644		
2	T1PKS	1-33916	ECO-0501	24%
3	Terpene	60762-81886	Petrichorin A/ petrichorin B	4%
4	NRPS	104152-161622	Nostopeptolide A1/ nostopeptolide 1052	25%
5	Terpene	158389-179519	2-methylisoborneol	100%
6	Lanthipeptide—class ii	267257-290133		
7	NRPS	79360-122572	Maduropeptin	39%
8	NRPS	1-56131	Keratinimicin A/ keratinimicin B/keratinimicin C/keratinimicin D	45%
9	T1PKS	65420-137997	Butyrolactol A	73%
10	Thiopeptide	117365-141061		
11	T1PKS	51805-98206		
12	T1PKS	1-5010		
13	RiPP-like	146675-157490		
14	NRPS	1-37588	Albachelin	30%
15	Hydrogen cyanide	52481-62326	Aborycin	14%
16	Arylpolyene	45422-86573	Kinamycin	5%
17	RiPP-like	71054-81425		
18	T1PKS	1-4963		
19	Aminopolycarboxylic acid	78689-92064	[S,S]-EDDS	100%
20	T1PKS	102319-131743	Mediomycin A	32%
21	Ectoine	29523-39912	Ectoine	100%
22	Terpene	62094-77620	Geosmin	100%
23	T1PKS	1-4023		
24	Terpene	13392-34321	Isorenieratene	42%
25	RiPP-like	559-46387	Amycolamycin A/ Amycolamycin B	31%
26	PKS-like	44016-85149		
27	Redox cofactor	53904-75938	Lankacidin C	26%
28	Terpene	91494-112456	Isorenieratene	71%
29	NRPS	112516-231634	Polyketide	40%
30	T1PKS	1-28437	ECO-02301	39%
31	Terpene	75321-96448	Leucomycin	3%
32	Lanthipeptide—class iii	26008-48563	Ery-9/Ery-6/Ery-8/Ery-7/ Ery-5/Ery-4/Ery-3	100%
33	NAPAA	20581-67646	Pyridomycin	19%
34	NRP-metallophore	45618-104827	Mirubactin	78%
35	Lasso peptide	438023-460583	Albusnodin	50%
36	T1PKS	1-89942	Hexacosalactone A	9%
37	NRPS	1-26044	Albachelin	80%
38	NRPS-like	1-49530	Ristomycin A	61%

**Table 4:** Functional prediction and consistency analysis of Cluster-35-related genes.

Number	Gene Length (bp)	Coding Protein	Homologous Strain (of Bacteria)	Similarity (%)
ctg80_440	1143	TadA family conjugal transfer-associated ATPase	*Amycolatopsis roodepoortensis*	98.68
ctg80_441	744	Type II secretion system F family protein	*Amycolatopsis roodepoortensis*	95.95
ctg80_444	1146	XRE family transcriptional regulator	*Amycolatopsis* sp. EV170708-02-1	93.93
ctg80_447	606	GCN5-related N-acetyltransferase	*Amycolatopsis alba*	94.47
ctg80_448	744	Lasso peptide biosynthesis B2 protein	*Amycolatopsis alba*	91.24
ctg80_449	1821	Albusnodin/ikarugamycin family macrolactam cyclase	*Amycolatopsis* sp. EV170708-02-1	90.28
ctg80_450	141	Albusnodin family lasso peptide	*Amycolatopsis alba*	95.35
ctg80_453	612	DUF4254 domain-containing protein	*Amycolatopsis alba* DSM 44262	77.17
ctg80_455	864	Helix-turn-helix transcriptional regulator	*Amycolatopsis* sp. EV170708-02-1	94.77
ctg80_456	225	DUF397 domain-containing protein	*Amycolatopsis alba*	94.59
ctg80_457	465	DUF4411 family protein	*Saccharothrix variisporea*	48.34
ctg80_458	1209	ImmA/IrrE family metallo-endopeptidase	*Mycobacteriales bacterium*	55.70
ctg80_462	2217	FtsK/SpoIIIE domain-containing protein	*Kibdelosporangium banguiense*	76.75

**Table 5:** Activity analysis of the fermented crude extracts in millet media.

Target Bacterium	Inhibitory Circle Diameter	Icon
*Acinetobacter baumannii*	20.0 ± 2.0 mm	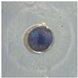
*Candida albicans*	13.0 ± 2.0 mm	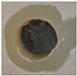
*Staphyloccocus aureus*	14.5 ± 2.0 mm	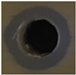
*Pseudomonas aeruginosa*	19.1 ± 2.0 mm	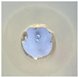
*Escherichia coli*	16.9 ± 2.0 mm	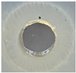

## Data Availability

The original contributions presented in this study are included in the article/Supplementary Materials. Further inquiries can be directed to the corresponding authors.

## Supplementary Materials

The following supporting information can be downloaded at: https://www.mdpi.com/article/10.3390/microorganisms14092096/s1,Table S1: Summary of Medium Names and Formulations; Table S2: Information of strains including species name, strain designation and GenBank accession number; Figure S1: Spectra of compound TRM77291-C2 ^13^C; Figure S2: ^1^H spectrum of compound TRM77291-C2.
