# Supplementary material for: Whole-Genome Analysis, Biosynthetic Gene Cluster Mining of Amycolatopsis sp. nov. TRM77291 and Antibacterial Activity of Siderochelin A
Source: Microorganisms. 2026 Sep 19;14(9):2096. doi: 10.3390/microorganisms14092096 (PMC13609835; doi:10.3390/microorganisms14092096)
Supplement: Supplementary file 1 [file microorganisms-14-02096-s001.zip › microorganisms-4543945-supplementary.pdf]

Support table S1 Summary of Medium Names and Formulations

1. Medium Name	Formulation
Gaoshi No.1 Medium	Soluble starch 20.0 g, potassium nitrate 1.0 g, dipotassium hydrogen phosphate 0.5 g, magnesium sulfate heptahydrate 0.5 g, ferrous sulfate 0.1 g, agar 16.0 g, distilled water 1000 mL, pH 7.2–7.4
MHA Medium	Beef extract 2.0 g, peptone 17.5 g, starch 2.0 g, agar 17.0 g, distilled water 1000 mL, pH 7.2–7.4
AM6 Medium	Starch 20.0 g, glucose 10.0 g, peptone 5.0 g, yeast extract 5.0 g, calcium carbonate 5.0 g, pH 7.2
Fermentation Medium X	Starch 30.0 g, glucose 20.0 g, soybean flour 22.0 g, rapeseed oil 6.0 g, sodium chloride 2.0 g, calcium carbonate 2.0 g, agar 16.0 g, distilled water 1000 mL, pH 7.2
MM Medium	Glucose 10.0 g, magnesium sulfate 0.2 g, ferrous sulfate 0.01 g, ammonium sulfate 1.0 g, dipotassium hydrogen phosphate 0.5 g, agar 16.0 g, distilled water 1000 mL, pH 7.2
Millet Medium	Boiled millet 15.0 g, glucose 5.0 g, peptone 4.0 g, sodium chloride 3.0 g, agar 16.0 g, distilled water 1000 mL, pH 7.0
ISP2 Medium	Yeast extract 4.0 g, malt extract 10.0 g, glucose 4.0 g, agar 20.0 g, agar 16.0 g, distilled water 1000 mL, pH 7.0
YEME Medium	Yeast extract 3.0 g, peptone 5.0 g, malt sugar 3.0 g, glucose 10.0 g, agar 16.0 g, distilled water 1000 mL, pH 7.0
AFMS Medium	Glucose 20.0 g, yeast extract 2.0 g, soybean flour 2.0 g, calcium carbonate 4.0 g, sodium chloride 1.0 g, agar 16.0 g, distilled water 1000 mL, pH 7.0
Modified GLY Medium	Glycerol 7.9 mL, lactose 5.0 g, malt extract 2.5 g, yeast extract 2.5 g, calcium carbonate 0.5 g, agar 16.0 g, distilled water 1000 mL, pH 7.0
SM13 Medium	Yeast powder 10.0 g, glycerol 25 mL, beef powder 10.0 g, peptone 5.0 g, magnesium sulfate heptahydrate 5.0 g, calcium carbonate 3.0 g, agar 16.0 g, distilled water 1000 mL, pH 7.4
Oatmeal-Soybean Flour Medium	Oatmeal 20.0 g, soybean flour 10.0 g, dipotassium hydrogen phosphate 1.5 g, magnesium sulfate heptahydrate 2.0 g, sodium chloride 3.0 g, agar 16.0 g, distilled water 1000 mL, pH 7.0

Support table S2. Information of strains including species name, strain designation and GenBank accession number

Name	Strain	Accession
Amycolatopsis speibonae	JS72	KF771257
Amycolatopsis lurida	DSM 43134	AJ577997
Amycolatopsis thailandensis	JCM 16380	NMQT01000085
Amycolatopsis roodepoortensis	M29	KF771262
Amycolatopsis coloradensis	DSM 44225	MQUQ01000038
Amycolatopsis umgeniensis	UM16	DQ110876
Amycolatopsis pittospori	PIP199	GU434264
Amycolatopsis decaplanina	DSM 44594	AJ508237
Amycolatopsis alba	DSM 44262	KB913032
Amycolatopsis azurea	DSM 43854	ANMG01000133
Amycolatopsis regifaucium	GY080	LQCI01000022
Amycolatopsis orientalis	DSM 40040	ASJB01000052
Amycolatopsis oliviviridis	SCM_MK2-4	LC225637
Amycolatopsis japonica	MG417-CF17	CP008953
Amycolatopsis lexingtonensis	NRRL B-24131	MUMJ01000484
Amycolatopsis benzoatilytica	AK 16/65	ARPK01000002
Amycolatopsis rifamycinica	DSM 46095	AY083603
Amycolatopsis camponoti	A23	KY952635
Amycolatopsis australiensis	DSM 44671	FPJG01000006
Amycolatopsis vastitatis	H5	LT621741
Amycolatopsis pretoriensis	DSM 44654	jgi.1085903
Amycolatopsis nigrescens	CSC17Ta-90	ARVW01000001
Amycolatopsis jiguanensis	CFH S01580	KU646989

Name	Strain	Accession
Amycolatopsis silviterrae	C12CA1	KR818707
Amycolatopsis mediterranei	IMSNU 20056	AJ293754
Amycolatopsis halotolerans	N4-6	DQ000196
Amycolatopsis echigonensis	LC2	AB248535
Amycolatopsis magusensis	KT2025	HQ157190
Amycolatopsis niigatensis	LC11	AB248537
Amycolatopsis rubida	DSM 44637	jgi.1085901
Amycolatopsis minnesotensis	32U-2	DQ076482
Amycolatopsis circi	S1-3	HQ021202
Amycolatopsis dendrobii	DR6-1	LC575120
Amycolatopsis equina	SE(8)3	HQ021204
Amycolatopsis hippodromi	S3-6	HQ021203
Amycolatopsis rhabdoformis	SB026	KF779477
Amycolatopsis xylanica	CPCC 202699	FJ529702
Amycolatopsis vancoresmycina	DSM 44592	AJ508240
Amycolatopsis jejuensis	NRRL B-24427	JNYZ01000077
Saccharothrix syringae	NRRL B-16468	JNYO01000115
Saccharothrix tamanrassetensis	SA198	JN036396
Saccharothrix saharensis	SA152	FJ379333
Saccharothrix coeruleofusca	NRRL B-16115	AF114805
Saccharothrix carnea	NEAU-yn17	KC195859
Saccharothrix australiensis	DSM 43800	RBXO01000001
Saccharothrix espanaensis	DSM 44229	HE804045
Saccharothrix variisporea	DSM 43911	RBXR01000001
Saccharothrix algeriensis	NRRL B-24137	AY054972
Saccharothrix luteola	NEAU-S10	OL378195
Saccharothrix yanglingensis	Hhs.015	GQ284639
Saccharothrix longispora	NRRL B-116116	AF114809
Saccharothrix ecbatanensis	UTMC 537	JN038074
Saccharothrix deserti	BMP B8144	MF972516
Saccharothrix texasensis	DSM 44231	RJKM01000001
Saccharothrix mutabilis subsp. capreolus	DSM 40225	X76965
Saccharothrix obliqua	SC076	LC634111
Saccharothrix violaceirubra	NBRC 102064	AB284261

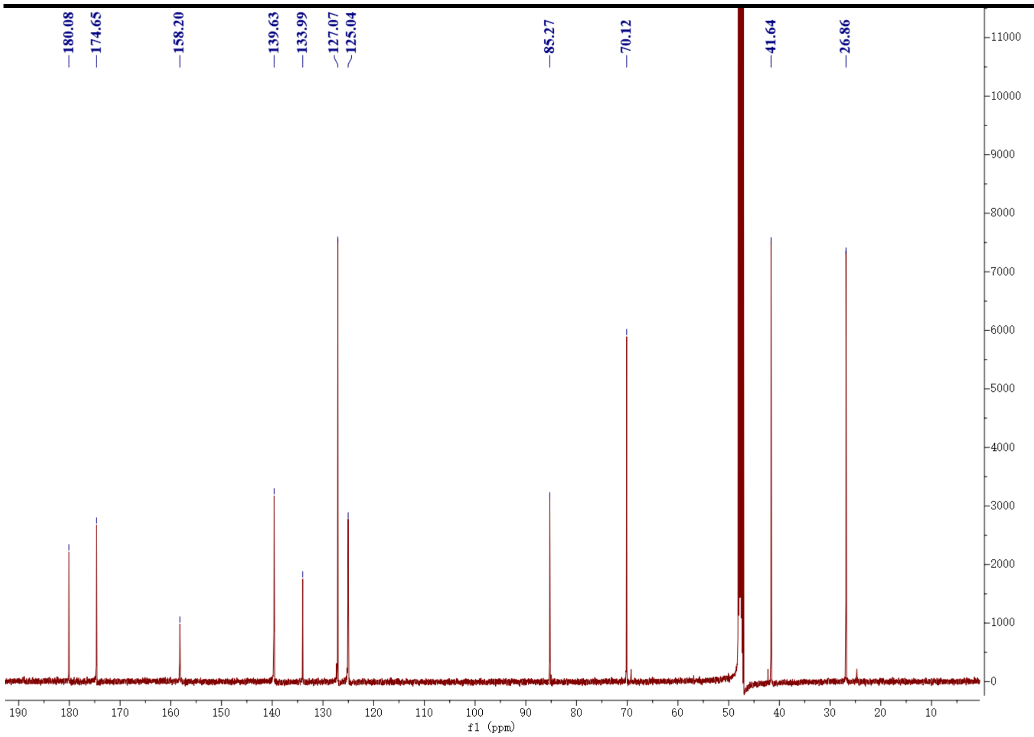

Support figure S1 Spectra of compound TRM77291-C2 ^{13}C

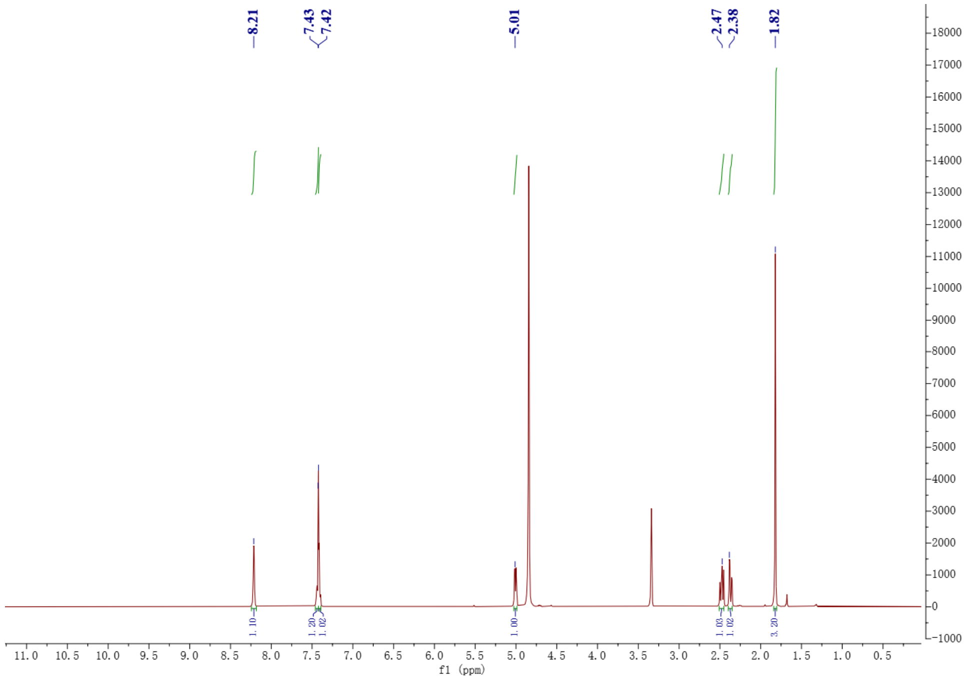

Support figure S2 ^{1}H spectrum of compound TRM77291-C2
